# Early prediction of the risk of scoring lower than 500 on the COMLEX 1

**DOI:** 10.1186/s12909-021-02501-5

**Published:** 2021-01-21

**Authors:** Qing Zhong, Han Wang, Payton Christensen, Kevin McNeil, Matthew Linton, Mark Payton

**Affiliations:** 1 Rocky Vista University College of Osteopathic Medicine, 255 E. Center St, Ivins, UT 84738 USA; 2 Shenzhen DJI Sciences and Technologies Ltd, Shen Zhen, China; 3 Rocky Vista University College of Osteopathic Medicine, Ivins, USA

**Keywords:** COMLEX 1: the comprehensive osteopathic medical licensing examination of the United States level 1, MCAT: the medical college admissions test, Cardiovascular system course, Renal system course, Respiratory system course, Predictive model, Score lower than 500 on COMLEX 1

## Abstract

**Background:**

The Comprehensive Osteopathic Medical Licensing Examination of the United States Level 1 (COMLEX 1) is important for medical students to be able to graduate. There is a glaring need to identify students who are at a significant risk of performing poorly on COMLEX 1 as early as possible so that extra assistance can be provided to those students. Our goal is to produce a reliable predictive model to identify students who are at risk of scoring lower than 500 on COMLEX 1 at the earliest possible time.

**Methods:**

Academic data from medical students who matriculated at Rocky Vista University College of Osteopathic Medicine between 2011 and 2017 were obtained. Odds ratios were used to assess the predictors for scoring lower than 500 on COMLEX 1. Correlation with COMLEX 1 scores was assessed with Pearson correlation coefficient. The predictive models were developed by multiple logistic regression, backward logistic regression, and logistic regression with average scores in courses in the first three semesters, and were based on performances on the Medical College Admissions Test (MCAT) before admission, as well as students’ performances in preclinical courses during the first three semesters. The models were generated in about 82% of the student performance data and were then validated in the remaining 18% of the data.

**Results:**

Odds ratios showed that MCAT scores and final grades in each course in the first three semesters were significant in predicting a score lower than 500 on COMLEX 1. Performances in third-semester courses including Renal System II, Cardiovascular System II, and Respiratory System II were most important in prediction. The three predictive models had sensitivities of 65.8 -71%, and specificities of 83.2 - 88.2% in predicting a score lower than 500 on COMLEX 1.

**Conclusions:**

Lower MCAT scores and lower grades in the first three semesters of medical school predict scoring lower than 500 on COMLEX 1. Students who are identified at risk by our models will have a 65.8 -71% chance of actually scoring lower than 500 on COMLEX 1. Those students will have enough time to receive assistance before taking COMLEX 1.

## Introduction

Students enrolled in an osteopathic medical school must pass the Comprehensive Osteopathic Medical Licensing Examination of the United States Level 1 (COMLEX-USA Level 1 or COMLEX 1) to be eligible to enroll in third year clinical rotations at some schools, to be qualified to take COMLEX-USA Level 2, and eventually to graduate and receive a Doctor of Osteopathic Medicine (DO) degree. Mitsouras et al. have observed a 4.8% rate of first-attempt failure on the COMLEX 1 among 1726 students at Western University of Health Science between 2010 and 2017 [1]. There is clear motivation for osteopathic medical schools to early identify those students who are at significant risk of failing or performing poorly on the COMLEX 1, so that extra assistance can be provided to those students through a variety of academic support channels.

Although some studies have been done to predict United States Medical Licensing Examination (USMLE) Step 1 or Step 2 exam performance for medical students, there are currently only a few studies that have attempted to predict COMLEX 1 performance from various preadmission and postadmission academic data. The COMLEX-USA examination is comparable to the allopathic licensing examination (USMLE) [2]. Preadmission variables that have been shown to positively correlate with COMLEX 1 score include undergraduate science grade point average (sciGPA) and Medical College Admission Test (MCAT) score [2–4]. Very high scores on the MCAT are also correlated with a COMLEX I score of 600 (80th percentile) or higher [5]. Postadmission variables, including performance in first-year and second-year medical school courses, predict scores on COMLEX 1 as well [2, 4–6]. In one study of 2146 students, all students in the top 20% of the class pass the COMLEX 1 on the first attempt, whereas only 64% of students who are ranked in the lowest 5% in the class pass [7]. Performance in the subject of pharmacology in an osteopathic medical school curriculum has also been found to strongly correlate with performance on COMLEX 1 [8]. For the last 20 years, many allopathic medical schools, as well as osteopathic medical schools, have implemented an organ-system based curriculum, but studies that have specifically connected an organ-system based curriculum and COMLEX 1 performance are rare [9]. Glaros et al. have found that the highest correlation with scores on COMLEX 1 is second semester Renal section course among all the courses in the first 2 years, as shown in one particular study of traditional organ-system curriculum [9]. Our school, Rocky Vista University College of Osteopathic Medicine, has a modified two-pass organ-system curriculum, which was initiated in 2011. Currently, it appears that there is no research studying the correlation between students’ performances in the modified organ-system courses and COMLEX 1, a gap in the literature that we are hoping to fill.

To help all students achieve success, it is critical to identify students at risk of poor performance on COMLEX 1 early. A score of 500 on COMLEX 1 has been regarded as national average for many years, but actually the percentile corresponding to a score of 500 has gradually decreased since 2011, from 43th in 2011 to 36th in 2020, according to the National Board of Osteopathic Medical Examiners (NBOME). A score of 500 on COMLEX 1 is truly below national average. Since the failing score is 400, a student who scores lower than 500 on COMLEX 1 has high risk of failing COMLEX 1. The purposes of our current project are to investigate the risk factors and generate reliable predictive models to identify students at the end of their third-semester who are at risk of performing lower than 500 on COMLEX 1. As a result, those students will have at least 7 months to get extra assistance before they must take COMLEX 1. We hope that the early intervention will enhance at risk students’ performance on COMLEX 1 and will help them avoid failing this examination.

## Methods

### Curriculum

Rocky Vista University College of Osteopathic Medicine (RVUCOM) is in the United States, and has a modified systems-based curriculum, which requires students to cover each system twice, once in the first year and again in the second year. This two pass, stepwise curriculum focuses on normal structure and function in the first year and transitions to abnormal function in the second year, with increased emphasis on pathology, pharmacology, and clinical application. The majority of the coursework of the first three semesters (first 1.5 years) is shown in Table 1.

**Table 1:** Odds Ratio with COMLEX-1 Score 500 as Cutoff (*N* = 904)

		Odds ratio	5% Conf. Lower	95% Conf. Upper	*P*
**Preadmission**	MCAT (first time)	0.925	0.876	0.977	**
	MCAT (mean)	0.912	0.858	0.972	**
**Semester I**					
Musculoskeletal System I	MSK I	0.984	0.981	0.988	***
Molecular Cellular Mechanism	MCM	0.985	0.981	0.988	**
Hematology/Immunology I	HEME I	0.988	0.985	0.991	***
Cardiovascular System I	CV I	0.983	0.980	0.987	***
Respiratory System I	RESP I	0.983	0.979	0.987	***
Renal System I	REN I	0.986	0.982	0.989	***
Osteopathic Principles/Practices I	OPP I	0.986	0.983	0.990	***
Principles of Clinical Medicine I	PCM I	0.986	0.982	0.990	***
**Semester II**					
Gastrointestinal System I	GI I	0.983	0.979	0.986	***
Endocrine/Reproductive System I	ENDO I	0.980	0.977	0.984	***
Neuroscience System I	NEURO I	0.982	0.979	0.986	**
Osteopathic Principles/Practices II	OPP II	0.984	0.980	0.987	***
Principles of Clinical Medicine II	PCM II	1.014	1.002	1.027	*
**Semester III**					
Renal System II	REN II	0.973	0.969	0.978	***
Cardiovascular System II	CV II	0.971	0.967	0.977	***
Respiratory System II	RESP II	0.976	0.972	0.979	***
Hematology/Lymphatic System II	HEME II	0.981	0.978	0.984	***
Gastrointestinal System II	GI II	0.979	0.976	0.983	***
Osteopathic Principles/Practices III	OPP III	0.981	0.978	0.985	***
Principles of Clinical Medicine III	PCM III	0.979	0.975	0.983	***
	Average score in all courses	0.955	0.950	0.964	***

Note: *** *p* < 0.0001; ** *p* < 0.01; * *p* < 0.05

*Abbreviations*: *COMLEX 1* The Comprehensive Osteopathic Medical Licensing Examination of the United States Level 1, *MCAT* the Medical College Admissions Test, *5% Conf. Lower* 5% confidence, *95% Conf. Upper*: 95% Confidence

### Participants

Our research project of “Using Simulation Modeling to Predict Failure on COMLEX 1 and 2 at First Attempt Through a Longitudinal Investigation” was approved by IRB committee of Rocky Vista University College of Osteopathic Medicine (RVUCOM), and the IRB number was IRB #2019-0079. The waiver was obtained for informed consent from IRB committee of RVUCOM since IRB determined the study was exempt. Academic performance data from seven cohorts (2011 to 2017) of students matriculated at Rocky Vista University College of Osteopathic Medicine were obtained. Students’ data were de-identified by the University registrar before disclosure to the investigators.

### Independent variables

Preadmission MCAT (the old version of the exam administered between 1991 and 2014) scores, and postadmission grades in each course in the first three semesters were used in this study. For students who took the MCAT more than once, average scores on all MCAT attempts were used. Scores on the MCAT from 1991 to 2014 ranged from a minimum of 3 to a maximum of 45. The 50th percentile was around 25. Scores in each course at RVUCOM were on a 1000 point scale reported in students’ transcripts.

### Dependent variable

De-identified COMLEX 1 scores on the first attempt for 904 students were collected.

### Statistical analysis

#### Univariate logistic regression

Univariate logistic regression was used to generate odds ratios. Dependent outcome was score on COMLEX 1. All independent variables were included. A score of 500 or higher was set to 0, and a score of 500 lower was set to 1. Threshold probability for positive classification was 0.5.

#### Bivariate correlations between variables

Pearson correlation coefficient (R) was used to measure the correlations between independent variables and dependent variable (score of COMLEX 1), and the correlations between each independent variable.

### Data for establishing and validating predictive models

Among 904 participants, some of the course grades were missing from the data. The 885 participants with complete data were randomly separated into a training data set with 728 participants (about 82%), and a testing data set with remaining 157 participants (about 18%). The models were developed with the training data set and were validated in the testing data set.

#### Model 1: multiple logistic regression

Independent variables included MCAT scores, and scores in each course in the first-three semesters. Dependent variable was score on COMLEX 1. A COMLEX 1 score ≥ 500 was set as 0, and a score of COMLEX 1 < 500 was set as 1. All independent variables were included in the final formula. Two cutoff probability values were tried and compared to find a better cutoff value.

A cutoff probability value of 0.5 was tried first. A student with a predicted probability equal to or higher than 0.5 is predicted to score below 500 on COMLEX 1, and a student with a predicted probability lower than 0.5 is expected to score 500 or higher on COMLEX 1.

A cutoff probability value of 0.25 was chosen later. A student with a predicted probability equal to or higher than 0.25 is predicted to score below 500 on COMLEX 1, and a student with a predicted probability lower than 0.25 is expected to score 500 or higher on COMLEX 1.

The sensitivity and specificity of prediction were compared between these two cutoff probability values, and cutoff probability value of 0.25 led to better accuracy in predicting the fraction of participants who scored lower than 500 on COMLEX 1. Therefore, the next two models were used with a cutoff probability value of 0.25 directly.

#### Model 2: backward stepwise logistic regression

Independent variables included MCAT scores, and scores in each course in the first three semesters. Dependent variable was score on COMLEX 1. We set a COMLEX 1 score ≥ 500 as 0, and < 500 as 1. Insignificant independent variables were removed sequentially until all variables were significant. The final formula contained only significant variables. A cutoff probability value of 0.25 was selected. A student with a predicted probability equal to or higher than 0.25 is predicted to score below 500 on COMLEX 1, and a student with a predicted probability lower than 0.25 is expected to score 500 or higher on COMLEX 1.

#### Model 3: logistic regression with average scores in all courses

In Model 3, the average score across all courses in the first three semesters was calculated for each student. This average score was used as a single independent variable in a logistic model. Dependent variable was score on COMLEX 1. We set a COMLEX 1 score ≥ 500 as 0, and < 500 as 1. As mentioned above, a cutoff value of 0.25 of probability was used; A student with a predicted probability equal to or higher than 0.25 is predicted to score below 500 on COMLEX 1, and a student with a predicted probability lower than 0.25 is expected to score 500 or higher on COMLEX 1.

For each predictive model, the number of true positives (TP) (participants who were predicted to have a score lower than 500 on COMLEX 1 who actually had a score lower than 500), false negatives (FN) (participants who were predicted to have a score of 500 or higher who actually had a score lower than 500), true negatives (TN) (participants who were predicted to have a score of 500 or higher who actually scored 500 or higher), and false positives (FP) (participants who were predicted to have a score lower than 500 who actually scored 500 or higher) were determined. Sensitivity (TP/(TP + FN)), and specificity (TN/(TN + FP)) were calculated.

All analyses were run using either IBM SPSS (Version 20, IBM SPSS Statistics, Chicago, IL), SigmaPlot 14 (Systat Software Inc., San Jose, CA), or SAS version 9.4 (SAS Institute, Cary, NC). The receiver operating characteristic curve (ROC) and the probability success plot were generated with Python language in the testing data set.

## Results

### Odds ratios of independent variables on a COMLEX 1 score lower than 500

To investigate the prediction with each independent variable on a score of less than 500 on COMLEX 1, odds ratios were generated by applying logistic regression to each independent variable. The odds ratios for all independent variables were shown in Table 1. Lower scores in the MCAT, each course (except PCM II (Principles of Clinical Medicine II)), and average scores in all courses in the first three semesters were all significant in predicting a COMLEX 1 score lower than 500. For example, the odds ratio for Cardiovascular System course (CV II) was 0.971, which meant that a 1-point decrease in a CV II score (on a 1000-point scale) will yield a 2.9% increase in odds of scoring lower than 500 on the COMLEX 1. Alternatively, for each 10-point (1%) reduction in a CV II score, the odds of getting a COMLEX 1 score of 500 lower will increase by 29%. Similarly, the odds ratio for the average score in all courses was 0.955, which meant that 1-point reduction in the average score in all courses, the odds of getting a COMLEX 1 score lower than 500 will increase by 4.5%. On the other hand, for each additional 10-point (1%) in the average score, the odds of performing higher than 500 on COMLEX 1 will increase by 45%. Each course score was on a 1000-point scale throughout this study.

### Bivariate correlation between independent variables and scores of COMLEX 1

As shown in Table 2, COMLEX 1 scores had a weak positive correlation with MCAT scores with a Pearson R of 0.18 (*p* < 0.05). COMLEX 1 scores had moderate-high positive correlation with all course scores, ranging from 0.41 to 0.7 (*p* < 0.05). The correlations with COMLEX 1 scores were gradually increased throughout our first three semester preclinical courses, if two clinical courses of OPP (Osteopathic Principles/Practices) and PCM (Principles of Clinical Medicine) were not considered. The third semester Renal System (REN II) and Cardiovascular System II (CVII) had the highest correlation with COMLEX 1, with a Pearson R of 0.7. In addition, course scores were significantly positively correlated with each other, ranging from 0.29 to 0.77 (*p* < 0.05). The correlations with MCAT scores were weak with first semester courses, ranging from 0.07 to 0.24, and were much weaker with the second and third semester courses, ranging from 0 to 0.12.

**Table 2:** Bivariate Correlation of Independent Variables and Scores of COMLEX 1 in the First Three Semesters (*N* = 904)

	COMLEX −1	MCAT	MSK I	MCM	HEME I	CV I	RESP I	REN I	OPP I	PCM I	GI I	ENDO I	NEURO I	OPP II	PCM II	REN II	CV II	RESP II	HEME II	GI II	OPP III	PCM III
**COMLEX-1**	**1.00**																					
**MCAT**	0.18	**1.00**																				
**MSK I**	0.54	0.14	**1.00**																			
**MCM**	0.49	0.24	0.60	**1.00**																		
**HEME I**	0.46	0.14	0.53	0.61	**1.00**																	
**CV I**	0.53	0.22	0.70	0.57	0.54	**1.00**																
**RESP I**	0.51	0.15	0.49	0.47	0.51	0.52	**1.00**															
**REN I**	0.55	0.15	0.55	0.53	0.47	0.60	0.56	**1.00**														
**OPP I**	0.41	0.09	0.60	0.42	0.43	0.57	0.51	0.47	**1.00**													
**PCM I**	0.41	0.07	0.49	0.44	0.45	0.50	0.45	0.36	0.50	**1.00**												
**GI I**	0.53	0.08	0.70	0.52	0.53	0.66	0.50	0.56	0.56	0.48	**1.00**											
**ENDO I**	0.61	0.12	0.66	0.54	0.55	0.62	0.58	0.60	0.54	0.49	0.67	**1.00**										
**NEURO I**	0.59	0.06	0.74	0.50	0.48	0.64	0.49	0.58	0.52	0.41	0.69	0.70	**1.00**									
**OPP II**	0.50	0.11	0.58	0.39	0.38	0.53	0.42	0.48	0.56	0.50	0.56	0.56	0.59	**1.00**								
**PCM II**	0.43	0.02	0.50	0.40	0.37	0.47	0.41	0.40	0.47	0.59	0.52	0.55	0.52	0.53	**1.00**							
**REN II**	0.70	0.09	0.51	0.48	0.49	0.55	0.53	0.57	0.43	0.44	0.58	0.65	0.62	0.53	0.52	**1.00**						
**CV II**	0.70	0.06	0.56	0.46	0.50	0.59	0.53	0.55	0.48	0.47	0.56	0.65	0.64	0.55	0.50	0.77	**1.00**					
**RESP II**	0.66	0.02	0.43	0.39	0.45	0.44	0.52	0.51	0.43	0.42	0.49	0.60	0.55	0.45	0.46	0.72	0.70	**1.00**				
**HEME II**	0.61	0.03	0.44	0.42	0.40	0.44	0.40	0.48	0.32	0.32	0.48	0.56	0.55	0.44	0.48	0.69	0.67	0.63	**1.00**			
**GI II**	0.63	0.02	0.46	0.39	0.42	0.48	0.47	0.52	0.38	0.41	0.52	0.61	0.60	0.48	0.52	0.73	0.71	0.72	0.71	**1.00**		
**OPP III**	0.49	0.00	0.45	0.32	0.29	0.36	0.37	0.38	0.41	0.35	0.41	0.45	0.50	0.53	0.49	0.51	0.52	0.50	0.53	0.54	**1.00**	
**PCM III**	0.49	0.06	0.44	0.34	0.35	0.43	0.36	0.37	0.36	0.50	0.46	0.51	0.45	0.52	0.57	0.55	0.58	0.48	0.51	0.56	0.48	**1.00**

Note: Pearson correlation *R* < 0.07, *p* > 0.05; Pearson correlation *R* ≥ 0.07, *p* < 0.05

*Abbreviations*: *COMLEX 1* The Comprehensive Osteopathic Medical Licensing Examination of the United States Level 1, *MCAT* the Medical College Admissions Test, *MSK I* Musculoskeletal System I, *MCM* Molecular Cellular Mechanism, *HEME I* Hematology/Immunology I, *CV I* Cardiovascular System I, *RESP I* Respiratory System I, *REN I* Renal System I, *OPP I* Osteopathic Principles/Practices I, *PCM I* Principles of Clinical Medicine I, *GI I* Gastrointestinal System I, *ENDO I* Endocrine/Reproductive System I, *NEURO I* Neuroscience System I, *OPP II* Osteopathic Principles/Practices II, *PCM II* Principles of Clinical Medicine II, *REN II* Renal System II, *CV II* Cardiovascular System II, *RESP II* Respiratory System II, *HEME II* Hematology/Lymphatic System II, *GI II* Gastrointestinal System II, *OPP III* Osteopathic Principles/Practices III, *PCM III* Principles of Clinical Medicine III

### Logistic regression models

The formulas of three logistic regression models were shown in Table 3.

**Table 3:** Formula of Logistic Regression Models to Predict COMLEX 1

	Formula
**Multiple logistic regression**	Log (Odds) = 37.176 - (0.0507 * MCAT) - (0.00212 * MSK I) - (0.00423 * MCM) + (0.00681 * HEMEI) - (0.00574 * CVI) - (0.00506 * RESP I) + (0.00451 * REN I) - (0.00130 * OPP I) + (0.00121 * PCM I) + (0.00178 * GI I) - (0.00403 * ENDO I) - (0.00528 * NEURO I) + (0.00193 * OPP II) + (0.00739 * PCM II) - (0.0114 * REN II) - (0.00884 * CV II) - (0.00759 * RESP II) - (0.00207 * HEME II) + (0.000838 * GI II) - (0.00555 * OPP III) - (0.00690 * PCM III)
**Backward logistic regression**	Log (Odds) = 32.4857 - 0.00759 (CV I) - 0.0128 (CV II) - 0.00781(RESP II) - 0.0132 (REN II)
**Logistic regression of average scores**	Log (Odds) = 35.5518 - 0.0442 (average score in all courses)

*Abbreviations*: *MCAT* the Medical College Admissions Test, *MSK I* Musculoskeletal System I, *MCM* Molecular Cellular Mechanism, *HEME I* Hematology/Immunology I, *CV I* Cardiovascular System I, *RESP I* Respiratory System I, *REN I* Renal System I, *OPP I* Osteopathic Principles/Practices I, *PCM I* Principles of Clinical Medicine I, *GI I* Gastrointestinal System I, *ENDO I* Endocrine/Reproductive System I, *NEURO I* Neuroscience System I, *OPP II* Osteopathic Principles/Practices II, *PCM II* Principles of Clinical Medicine II, *REN II* Renal System II, *CV II* Cardiovascular System II, *RESP II* Respiratory System II, *HEME II* Hematology/Lymphatic System II, *GI II* Gastrointestinal System II, *OPP III* Osteopathic Principles/Practices III, *PCM III* Principles of Clinical Medicine III, *Average score in all courses* Average score of all first three semester courses

Multiple logistic regression model had 21 variables in the formula as shown in Table 3. As shown in Table 4, when using a cutoff probability value of 0.5, multiple logistic regression model yielded a sensitivity of 48.2% in the training data set, and of 44.7% in the testing data set. When the cutoff value was changed to 0.25, the sensitivity was increased to 75.9% in the training data set, and 68.4% in the testing data set. Additionally, with the cutoff value of 0.25, the testing data set had a specificity of 88.2%, which was close to the specificity of 92.4% with the cutoff value of 0.5. Therefore, all three models adopted cutoff probability value of 0.25 because of better accuracy of prediction.

**Table 4:** Logistic Regression Models for Detecting Students Scoring Less Than 500 on COMLEX 1

Model	Training data (***N*** = 728)		Testing data (***N*** = 157)	
	Sensitivity	Specificity	Sensitivity	Specificity
**Cutoff value of 0.5**				
Multiple logistic regression	82/170 (48.2%)	528/558 (94.6%)	17/38 (44.7%)	110/119 (92.4%)
**Cutoff value of 0.25**				
Multiple logistic regression	129/170 (75.9%)	475/558 (85.1%)	26/38 (68.4%)	105/119 (88.2%)
Backward logistic regression	135/170 (79.4%)	447/558 (80.1%)	25/38 (65.8%)	105/119 (88.2%)
Logistic regression of average scores	134/170 (78.8%)	430/558 (77.0%)	27/38 (71.0%)	99/119 (83.2%)

**Fig. 1:**
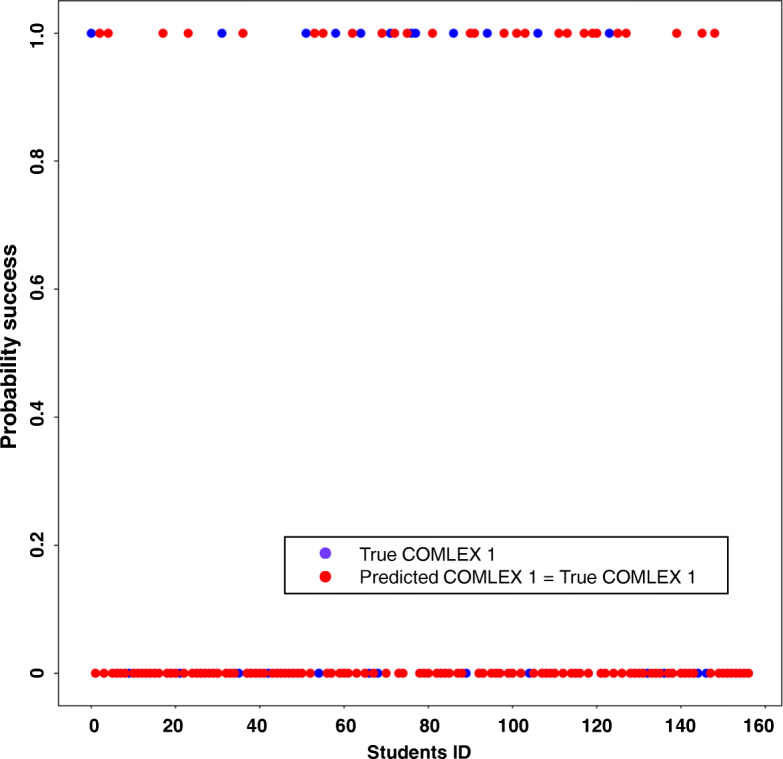
Probability Success Plot of Backward Logistic Regression Model. This is the validation of the backward logistic regression model in the testing data set. Probability of 1 means scores lower than 500 on COMLEX 1, and probability of 0 represents scores equal or higher than 500 on COMLEX 1. The blue dots represent the true COMLEX 1 scores, and red dots represent the predicted COMLEX 1 matched to true COMLEX 1 scores

As shown in Table 3, the backward stepwise logistic regression model had four significant variables left in the final formula. Scores in Cardiovascular System I (CV I), Cardiovascular System II (CV II), Renal System II (REN II) and Respiratory System II (RESP II) were significant in predicting COMLEX I scores lower than 500 in this model. As shown in Table 4, this model had a sensitivity of 79.4% and a specificity of 80.1% in training data set, which is comparable to the multiple logistic regression model. The reduced number of variables in this model did not decrease the accuracy of prediction. The prediction accuracy of backward logistic regression model was validated in the testing data set, which yielded a sensitivity of 65.8%, and a specificity of 88.2%. This was visualized in Fig. 1. Figure 1 showed at probability of 1 (corresponding to scoring lower than 500 on COMLEX 1), 25 out of 38 students who actually scored lower than 500 on COMLEX 1 were identified, and at probability of 0 (corresponding to scoring higher than 500 on COMLEX 1), 105 out of 119 students whose actual COMLEX 1 scores higher than 500 were detected.

As shown in Table 4, the logistic regression model with average scores in all courses identified 134 out of 170 participants who actually scored lower than 500 on COMLEX 1 (sensitivity 78.8%), and 430 out of 558 participants who really scored higher than 500 (specificity 77%) in the training data set. In the testing data set, this model had a sensitivity of 27/38 (71%), and a specificity of 99/119 (83.2%).

**Fig. 2:**
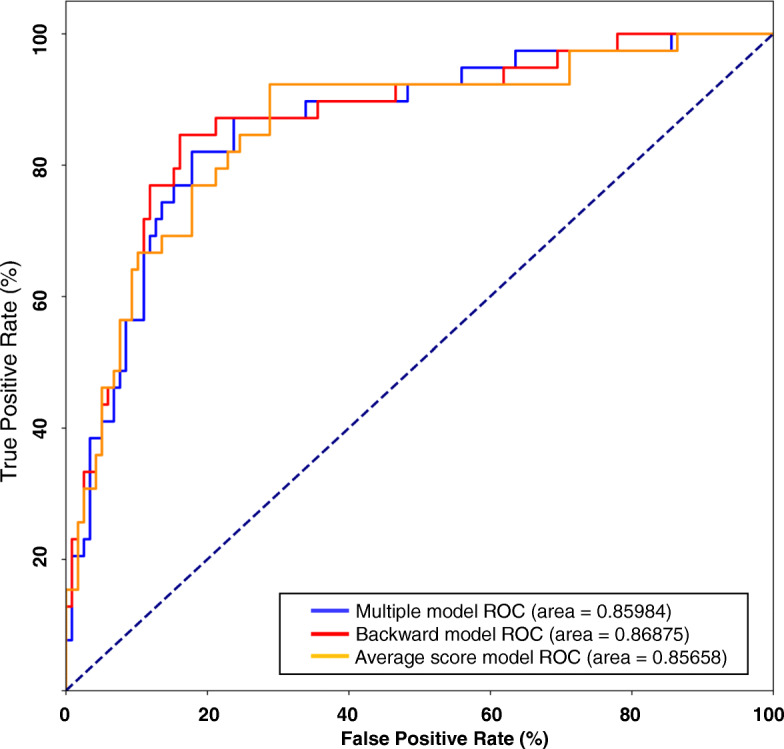
Receiver Operating Characteristic Curve (ROC) of Three Models. ROC measures the true positive rate and false positive rate at all values of probability. The area under the ROC curve evaluates model classification accuracy; the higher the area, the bigger the disparity between true and false positives, and thus the stronger the model in classifying members. The blue colored curve was for the multiple logistic regression model whose area under the curve was 0.85984. The red colored curve was the backward logistic regression model, which has an area under the curve of 0.86875. The yellow colored curve was for the logistic regression model with average scores in all courses, and its area under the curve was 0.85658

To compare the accuracy of prediction among the three models, the receiver operating characteristic curve (ROC) of the three Models is shown in Fig. 2. The ROC curves of the three models were overlaid on each other, and had very similar area ranging from 0.85658 to 0.86875. The backward logistic regression model had the largest area, and model of the logistic regression with average scores had the smallest area. Therefore, backward logistic regression model was the best to predict a COMLEX 1 score lower than 500.

## Discussion

Our study found that MCAT scores and scores in each course in the first three semesters were all significant in predicting COMLEX 1 scores lower than 500. The multiple logistic regression model, backward stepwise logistic regression model, and the logistic regression model with average scores identified 65.8 -71% of students who actually scored lower than 500 on COMLEX 1 at their first attempt.

From our results, a low MCAT score was a weak but significant predictor of scoring lower than 500 on COMLEX 1. This is consistent with the literature, in which MCAT scores have been found to positively correlate with COMLEX 1 performance [3, 4, 10]. Additionally, Vora et al. have found that students with COMLEX 1 scores of 600 (80th percentile) or higher are 1.3 times more likely to have a higher MCAT score [5]. Similarly, Gauer et al. have demonstrated that a MCAT score lower than 28 (66.8th percentile) predicts a USMLE Step 1 score lower than 207 (12th percentile), and a MCAT score higher than 40 (99.8th percentile) predicts a USMLE Step 1 score of 260 (96th percentile) or higher [11]. Our study and the literature provide evidence that the MCAT score is still an important criterion for selecting medical student candidates, in terms of predicting success on standardized board examinations.

Lower performance in each course except PCM II course in the first three semesters at RVUCOM was a predictor of a COMLEX 1 score lower than 500 in our study. Among all courses, Renal System II Course (REN II), Cardiovascular System I (CV I) and II Courses (CV II), and Respiratory System II Course (RESP II) were the strongest predictors, according to the correlation coefficients and the backward stepwise logistic regression. Similar to our findings, Glaros et al. also have identified that the second semester Renal section course is the number one predictor for COMLEX 1 scores among all preclinical courses in a traditional organ system curriculum at their institution [9]. In our study, Renal System II (REN II), Cardiovascular System II (CV II), and Respiratory System II (RESP II) are courses in the third-semester. REN II course is implemented at the beginning of the second year, and is followed by CVII and RESP II. It seems that course performance in the third semester, at the beginning of the second year, is most important in predicting COMLEX 1 scores lower than 500. There is currently no explanation in the literature as to why these courses are so important for performance on COMLEX 1. The authors postulate that renal, cardiovascular, respiratory system courses involve understanding and heavy integration of anatomy, physiology, pathology, and pharmacology, all of which are heavily tested on COMLEX 1.

To predict low performance on COMLEX 1 early, we developed three models: multiple logistic regression, backward stepwise logistic regression and logistic regression with average scores in all course in the first three semesters. The three models had very close sensitivities and specificities. Sensitivity and specificity for each model were similar between the training data set and the testing data set. This means that each model is reliable for prediction of COMLEX scores lower than 500 and identification of students at risk. Among the three models, the backward logistic regression model was the best in term of accuracy of prediction. Because course scores were positively correlated with each other, backward logistic regression was better than multiple logistic regression to minimize the influence of collinearity. According to our models, if a student is predicted by our models to score lower than 500 on COMLEX 1, this student will have a 65.8 -71% chance that he or she will actually score lower than 500. Once students are identified to be at risk of a poor COMLEX 1 performance, those students will still have at least 7 months (5 months of the fourth-semester, plus two or three more months to prepare) before taking COMLEX 1. Thus, they will have time to adjust their study patterns and to focus on the content they need to master. Also, schools will have time to provide extra assistance to help those students.

To our knowledge, our study is the first to use the first three semesters of preclinical courses to predict a COMLEX 1 score lower than 500. Compared to any other models built at the end of the second year or after the fourth semester, our current models have the advantage of letting students who are at risk of poor performance on COMLEX 1 have enough time to modify their study strategy and receive assistance before they must take COMLEX 1.

Our study has limitations. Our study used scores from the old MCAT, the 1991-2014 version of the test. Since 2015, the new MCAT has gradually replaced the old MCAT, and the score scaling is different on the new exam. Therefore, to compare an old MCAT score with a new MCAT score, the same percentile can be used [12]. Students in the 50th percentile received a score of approximately 25 in old MCAT scores, which is comparable to 500 in new MCAT Scaled Scores [12]. In addition, other medical schools may have different curriculum than RVUCOM, so our predictive models may not apply to other medical schools. Some medical schools have converted to a pass/fail grading system recently [13], therefore our models may not work in schools with this new grading system.

In conclusion, lower MCAT scores and lower scores in preclinical courses are significant predictors of a COMLEX 1 score lower than 500. Performances on third semester courses including Renal System II, Cardiovascular System II, and Respiratory System II, are the top predictors of poor performance on COMLEX 1. Our three predictive models, based on MCAT scores and student performance in courses in the first three semesters, have similar accuracy in predicting poor performance on COMLEX 1, but the backward logistic regression model turns out to be the best among the three models. Our models have the advantage of early prediction, giving students enough time to better prepare for COMLEX 1. In the future, studies are needed to explore new predictive modeling using the new version of the MCAT and a new pass/fail grading curriculum.

## Data Availability

All data used in the study are only available for interested researchers upon request from the corresponding author after approval from the Institutional Review Board at RVU.

## Abbreviations

COMLEX 1: The Comprehensive Osteopathic Medical Licensing Examination of the United States Level 1
MCAT: The Medical College Admissions Test (MCAT)
SciGPA: Undergraduate science grade point average
RVUCOM: Rocky Vista University College of Osteopathic Medicine
CV I: Cardiovascular System I course
CV II: Cardiovascular System II course
REN II: Renal System II course
RESP II: Respiratory System II course
